# Comparing Cost-Effectiveness of HIV Testing Strategies: Targeted and Routine Testing in Washington, DC

**DOI:** 10.1371/journal.pone.0139605

**Published:** 2015-10-14

**Authors:** Amanda D. Castel, Sungwoog Choi, Avi Dor, Jennifer Skillicorn, James Peterson, Nestor Rocha, Michael Kharfen

**Affiliations:** 1 The Milken Institute School of Public Health at the George Washington University, Washington, DC, United States of America; 2 The George Washington University School of Public Policy and Public Administration, Washington, DC, United States of America; 3 District of Columbia Department of Health, HIV/AIDS, Hepatitis, STD, TB Administration, Washington, DC, United States of America; Emory University Rollins School of Public Health, UNITED STATES

## Abstract

**Background:**

Routine HIV testing is an essential approach to identifying undiagnosed infections, linking people to care and treatment, and preventing new infections. In Washington, DC, where HIV prevalence is 2.4%, a combination of routine and targeted testing approaches has been implemented since 2006.

**Methods:**

We sought to evaluate the cost effectiveness of the District of Columbia (DC) Department of Health’s routine and targeted HIV testing implementation strategies. We collected HIV testing data from 3 types of DC Department of Health-funded testing sites (clinics, hospitals, and community-based organizations); collected testing and labor costs; and calculated effectiveness measures including cost per new diagnosis and cost per averted transmission.

**Results:**

Compared to routine testing, targeted testing resulted in higher positivity rates (1.33% vs. 0.44%). Routine testing averted 34.30 transmissions per year compared to targeted testing at 17.78. The cost per new diagnosis was lower for targeted testing ($2,467 vs. $7,753 per new diagnosis) as was the cost per transmission averted ($33,160 vs. $104,205). When stratified by testing site, both testing approaches were most cost effective in averting new transmissions when conducted by community based organizations ($25,037 routine; $33,123 targeted) compared to hospitals or clinics.

**Conclusions:**

While routine testing identified more newly diagnosed infections and averted more infections than targeted testing, targeted testing is more cost effective per diagnosis and per transmission averted overall. Given the high HIV prevalence in DC, the DC Department of Health’s implementation strategy should continue to encourage routine testing implementation with emphasis on a combined testing strategy among community-based organizations.

## Introduction

Routine HIV testing is an essential tool to identify undiagnosed HIV infections, link people to care and treatment, reduce HIV-related morbidity and mortality, and prevent new infections. At least 20% of HIV-infected persons are estimated to be unaware of their HIV infection and account for almost half (49%) of new HIV transmissions [1, 2]. Reducing new infections by increasing awareness of one’s status and reducing transmission is one goal of the National HIV AIDS Strategy and is a critical component of population-based approaches such as treatment as prevention [3, 4].

In 2006 the Centers for Disease Control and Prevention (CDC) issued revised recommendations on the implementation of routine HIV testing in health-care settings [5]. The recommendations called for routine opt-out HIV testing to be offered in all health-care settings, including substance use treatment settings, correction healthcare facilities, emergency and urgent care clinics, primary care settings, and all public health and community health-care clinics [5]. The recommendations were focused on areas where the HIV prevalence was greater than or equal to 0.1%. While the recommendations modified approaches to testing in health-care settings, they did not modify existing guidelines concerning HIV counseling, testing, and referral for high-risk persons who seek or receive HIV testing in non-clinical settings (e.g., community-based organizations, outreach settings, or mobile vans) [5].

Implementation of the 2006 CDC recommendations was facilitated by the advent of new testing technologies. With the availability of highly sensitive and specific rapid tests, testing expansion was feasible in both clinical and non-clinical settings. Rapid tests can be administered using oral swabs or fingersticks, and test results can be returned in as few as 20 minutes whereas in the past conventional testing required a blood draw and it often took several days to return test results.

In Washington, DC, 2.5% of the population is estimated to be living with HIV and as many as 30% of persons at high-risk for HIV are estimated to be unaware of their HIV infection [6–10]. Thus early implementation of the revised recommendations has been critical to improving diagnosis of HIV infection. In June 2006, the District of Columbia Department of Health (DC DOH) HIV/AIDS, Hepatitis, STD, TB Administration began implementing routine, opt-out HIV testing in health-care settings in anticipation of the September release of the CDC recommendations [11]. The initial DC testing initiative, “Come Together D.C.–Get Screened for HIV,” engaged community-based and clinical providers throughout the community to perform rapid HIV screening, launched extensive social marketing campaigns to educate residents and providers about routine HIV testing, and trained providers to facilitate immediate linkage to care for those with a positive HIV test [11]. This campaign laid the foundation for future HIV testing initiatives and programs in DC.

While the DC DOH has made routine, opt-out screening an integral part of its HIV prevention initiatives, in alignment with the CDC’s 2006 guidelines, the DC DOH has also continued to fund and support targeted HIV testing among high-risk populations [5]. Targeted HIV testing is critical to ensure that persons at high-risk (including African-Americans, injection drug users, and persons engaged in sex work) are aware of their HIV status. Although these combined efforts have proven successful in identifying HIV-infected persons and linking them to care, neither a robust programmatic evaluation nor a systematic analysis of the cost-effectiveness of these programs has been conducted to date [12]. Therefore, in 2011, as part of the Enhanced Comprehensive HIV Prevention Planning Initiative (ECHPP), a federal initiative to maximize HIV prevention activities in areas with the highest HIV prevalence in the US, researchers from the DC Developmental Center for AIDS Research worked in partnership with the DC DOH to evaluate the cost effectiveness of the DOH’s routine and targeted HIV testing implementation strategies [13, 14].

## Materials and Methods

For the purposes of this analysis routine HIV testing was defined as screening for HIV being conducted as per the CDC guidelines, which was defined as non-risk based, opt-out HIV testing for patients in all health care settings. This testing did not require separate written consent, assessment of risk behaviors, or prevention counseling, and testing was conducted unless a patient declined testing (opted-out)[5]. In contrast, targeted HIV testing was defined as HIV screening among persons at high risk for HIV based on an assessment of clinical, demographic or behavioral risk factors (e.g. injection drug use, multiple sexual partners, men who have sex with men).

Given the interest in evaluating specific routine and targeted testing programs, this cost-effectiveness analysis was designed from the payer’s perspective, incorporating the major components of direct costs [15]. The analysis takes into account that routine testing is performed primarily in clinical settings, on a non-risk basis, whereas targeted testing is performed in both clinical and non-clinical settings, focusing on high-risk persons only. Furthermore, different from prior approaches, we performed an ex-post cost-effectiveness analysis of routine HIV testing and targeted testing using actual testing results, testing costs, and other parameters as observed within program sites. Our data are specific for DC for FY2011 [16].

HIV testing data, including number of tests performed and test results, were provided from the DC DOH through their Program Evaluation and Monitoring System (PEMS). PEMS is a CDC-designed national electronic data reporting system used to track data on testing conducted by health departments and community providers that receive support through CDC HIV prevention cooperative agreements [17]. At the local level, PEMS systematically and routinely captures information on the facility performing the test, the type of testing being performed at the site (i.e., routine vs. targeted), the client’s unique identification number, preliminary test results, and information regarding referrals to HIV primary care. Data from all 46 DC DOH funded testing sites in PEMS were included in this analysis representing 20 community-based organizations, (CBOs), 18 clinics, and 8 hospitals. Information on the testing approach being used by each site was provided by DC DOH staff.

Following methodology used by Holtgrave et al (2007), our cost effectiveness measure was defined as the annual number of averted HIV transmissions, using nationally reported transmission rates for HIV-infected individuals [16]. The number of averted HIV transmissions was calculated using the difference in HIV transmission rate between people who know they are living with HIV/AIDS (Aware PLWHA) and people who do not know they are living with HIV (Unaware PLWHA) [16]. Unaware PLWHA transmit HIV at a higher annual rate than aware PLWHA [18]. We assumed 100% receipt of testing results regardless of testing type and that the transmission rate dropped after an unaware PLWHA becomes aware of his/her HIV status. Hence, the number of averted HIV transmissions was estimated by multiplying the number of unaware PLWHA and the difference in transmission rate before and after knowing one’s HIV status. We performed these calculations separately for routine and targeted testing.

$$A=N_{u}(T_{u}-T_{a})$$

where A is the number of averted HIV transmissions, N_u_ is the newly identified unaware PLWHAs, T_u_ is the average HIV transmission rate from unaware PLWHAs, and T_a_ is the average HIV transmission rate from aware PLWHAs.

To estimate the cost per averted HIV transmission, we considered a variety of input parameters including testing data, transmission rates, numbers of infections averted, and testing and labor costs. Following Holtgrave et al. (2012) the transmission rate of PLHIV who are unaware of their status was estimated at 10.20%, while that of aware PLWHAs was estimated at 2.76% [18].

HIV costing data were collected through interviews with DC DOH staff, surveillance reports, and document review. HIV costs were adjusted in 2010 US dollars, and the study period was for April 2010 –March 2011. The total cost of HIV testing was calculated using the sum of testing costs and labor costs. The total testing cost was derived by multiplying the number of HIV tests by unit price. The total number of HIV tests and their purchasing costs were collected from each site type and are reported in Table 1.

**Table 1:** Routine and Targeted Testing Costs by Site and Test Type.

Site Type	Test Type	Number of Sites	Routine		Targeted	
			Tests (#)	Testing Costs ($)	Tests (#)	Testing Costs ($)
**CBO**	**All**	**20**	**1,422**	**15,871**	**17,640**	**196,907**
	Oral	20	1,422	15,871	17,640	196,907
	Blood	0	0	0	0	0
	Finger	0	0	0	0	0
	Mixed	0	0	0	0	0
**Clinic**	**All**	**18**	**67,577**	**946,076**	**292**	**2,457**
	Oral	15	24,489	273,222	72	807
	Blood	1	1,512	11,340	0	0
	Finger	1	10,221	76,658	220	1,650
	Mixed	1	31,355	584,856	0	0
**Hospital**	**All**	**8**	**35,425**	**420,595**	**0**	**0**
	Oral	3	6,555	76,743	0	0
	Blood	0	0	0	0	0
	Finger	2	16,486	127,246	0	0
	Mixed	3	12,384	216,606	0	0
**Total**		**46**	**104,424**	**1,382,542**	**17,932**	**199,364**

Source: Program Evaluation and Monitoring System (PEMS), Fiscal year 2011 Washington, DC DOH.

Unit cost of different types of HIV testing are shown in Table A in S1 File. The DC DOH made a bulk purchase of rapid test kits which they provided to participating sites free of charge. The unit cost of each test kit was included. The various testing agencies relied on different testing technologies that required oral fluid, venipuncture, fingerstick blood, or combined use of oral fluid and fingerstick blood testing. For oral tests, we included the additional standard cost of a control test for each 100 test units. In addition, clinics and hospitals may perform supplementary tests (confirmatory Western Blot tests) to confirm seropositive results from general testing; CBOs generally do not have the capacity to conduct such tests their own. In order to compare a fixed bundle of services across agency types, we report cost effectiveness results without confirmatory Western Blot tests.

Since uniform budget or expense data were not available from the agencies in our study, we constructed estimates of labor costs by multiplying reported hours of work by the wage rate. Following Farnham et al. (2008), we divided labor effort of HIV testing into three categories: pre-test counseling, HIV antibody testing, and post-test counseling [19]. Our estimates are summarized in Table 2. Additional detail on hours and wages by agency type and labor category are shown in Table B in S1 File. Note that unlike clinics and CBOs, in our data hospitals did not provide pretest counseling. Information on hourly wage of counselors, administrative workers, and lab technicians in clinics and hospitals was obtained from Bureau of Labor Statistics wage data for the Washington DC metropolitan area; wage information for CBOs was directly collected from a sample of CBOs in DC (Table B in S1 File). Additionally, sites that were funded to conduct targeted testing were assumed to already have access to these higher risk populations, thus no additional costs were assumed for the identification of these targeted populations.

**Table 2:** Routine and Targeted Labor Costs by Site and Test Type.

Site Type	Number of Sites	Routine	Targeted
		Labor Costs ($)	Labor Costs ($)
CBO	20	30,697	382,209
Clinic	18	1,865,905	8,071
Hospital	8	294,917	0
Total	46	2,191,519	390,280

We calculated two distinct cost effectiveness ratios: costs per new diagnosis (C/N), and costs per averted diagnosis (C/A). Table 3 presents a comparison of findings for routine versus targeted testing. Table 4 further stratifies the results by facility type. Given the interest in gaining specific insights into on each of the programmatic strategies, i.e. routine and targeted testing, rather than recommending one strategy while abandoning the other, we focus on the cost effectiveness ratios themselves. However for illustrative purposes we also report the incremental cost effectiveness ratio (ICER), defined as ΔC/ΔE = C_b_−C_a_ / E_b_−E_a_ where C is cost, E is effectiveness (averted diagnosis), b indexes routine testing, and a indexes targeted testing.

**Table 3:** Cost Effectiveness of Routine and Targeted HIV Testing, Washington, DC, 2011.

Measure	Routine HIV testing	Targeted HIV testing
a. number of tests	104,424	17,932
b. number testing positive, unique	497	328
c. number testing positive, aware	36	89
d. number testing positive, unaware	461	239
e. portion of number testing positive, unaware	0.44%	1.33%
f. transmission rate from unaware HIV +	10.20%	10.20%
g. transmission rate from aware HIV +	2.72%	2.72%
h. number of transmissions averted^1^ ^,^ ^2^	34.48	17.88
i. testing costs ($)	1,382,542	199,364
j. labor cost ($)	2,191,519	390,280
k. total cost ($)	3,574,061	589,644
l. cost per new diagnosis ($)	7,753	2,467
m. cost per averted transmission ($)	103,648	32,983
Incremental CE ratio (ICER), per averted transmission	179,784	

^1)^ the number of averted HIV transmissions is estimated by multiplying the number of persons with HIV who became aware of their status and the difference in transmission rates before and after knowing their HIV status. h = d * [f–g].

^2)^ The average HIV transmission rate for all groups was used for the number of averted transmissions.

**Source**: Program Evaluation and Monitoring System (PEMS), Fiscal year 2011 Washington, DC DOH

**Table 4:** Cost Effectiveness of Routine and Targeted HIV Testing by Site Type, Washington, DC, 2011.

Measure	Routine			Targeted		
	CBO	Clinic	Hospital	CBO	Clinic	Hospital
a. number of tests	1,422	67,577	35,425	17,354	578	0
b. number testing positive, unique	25	311	161	324	4	0
c. number testing positive, aware	0	33	3	89	0	0
d. number testing positive, unaware	25	278	158	235	4	0
e. portion of number testing positive, unaware	1.76%	0.41%	0.45%	1.35%	0.69%	N/A
f. transmission rate from unaware HIV +	10.20%	10.20%	10.20%	10.20%	10.20%	10.20%
g. transmission rate from aware HIV +	2.72%	2.72%	2.72%	2.72%	2.72%	2.72%
h. number of transmissions averted	1.87	20.79	11.82	17.58	0.302	0
i. testing costs ($)	15,871	946,076	420,595	196,907	2,457	0
j. labor cost ($)	30,697	1,865,904	294,917	382,209	8,070	0
k. total cost ($)	46,568	2,811,980	715,513	579,116	10,528	0
l. cost per new diagnosis ($)	1,863	10,115	4,529	2,464	2,632	N/A
m. cost per averted transmission ($)	24,903	135,228	60,542	32,946	35,187	N/A

Source: Program Evaluation and Monitoring System (PEMS), Fiscal year 2011 Washington, DC DOH

Note that to assure that costs pertained to a standardized service across facility types, we excluded agency fixed costs, namely rent and maintenance expenditures as they could not be allocated to HIV-testing activities specifically. Thus, in an accounting sense, our analysis pertains to variable costs only.

The study was reviewed and approved by the DC Department of Health and George Washington University Institutional Review Boards.

## Results

In 2011, 122,356 DOH-funded HIV tests were performed in a combination of clinical and non-clinical sites in Washington, DC. Persons tested were 65% black, 50% male, and 49% were between the ages of 20 and 39. Most persons tested did not identify a high risk behavior for HIV, however, 37% identified as heterosexual and 2% as men who have sex with men (data not shown). Table 3 compares the testing results, effectiveness, and cost measures for routine versus targeted testing. In 2011, 104,424 routine HIV tests and 17,932 targeted HIV tests were performed by 46 HIV testing agencies in Washington, DC. Among the routine HIV tests, 461 persons (0.44%) were newly identified as HIV-positive compared to targeted testing in which 239 persons (1.33%) were newly diagnosed. With respect to transmissions averted, more transmissions per year were averted using routine testing compared to targeted testing (34.3 vs. 17.78); yielding a relatively modest 1.9 fold improvement over targeted testing.

Total costs for routine HIV testing in 2011 were estimated at $3,574,061; total costs for targeted HIV testing were substantially lower ($589,644 in total costs). Overall, the cost per new diagnosis was estimated to be $7,753 for routine testing while that of targeted testing was estimated at $2,467. Additionally, the cost per averted transmission was substantially higher for routine testing than targeted testing ($104,205 vs. $33,160) resulting in a CE ratio for targeted testing of less than one-third the CE ratio for routine testing.

A further analysis of testing results, effectiveness, and cost measures stratified by type of testing site is presented in Table 4. With routine testing, although clinics tested the largest number of people, the highest proportion of people testing newly positive was found among CBOs (1.76% positivity rate) and all people testing positive were previously unaware of their infection. Despite CBOs having the highest positivity, clinic-based routine testing yielded the most transmissions averted at 20.68, almost two times that of hospital-based routine testing (11.76) and more than 10 times that of CBO-based routine testing (1.86).

When examining the results from targeted testing, CBOs performed the most tests using this approach (n = 17,354), had the highest number and proportion of persons testing newly positive (n = 235, 1.35%) and the highest number of transmissions averted (17.48 transmissions per year) compared to clinic and hospital-based targeted testing. Testing, labor, and total costs were highest for clinics doing routine testing but highest for CBOs doing targeted testing. However, the cost per new diagnosis and cost per transmission averted using both testing approaches was lowest for CBOs at $1,863 and $2,464, respectively and $25,037 and $33,123 per transmission averted, respectively.

We performed several sensitivity analyses including adding in the costs of Western blot testing and using a different set of assumptions for differences in transmission risk. Adding the adding Western blot costs resulted in very minor changes, and did not affect the results overall (Table C and Table D in S1 File). When assessing differences in the assumed transmission rates after diagnosis, we found that when the portion of people with risky behaviors was doubled, the cost per averted transmission was $53,091, which is 57.1% higher than the previous cost effectiveness ratio for targeted testing (Table E in S1 File). Hence, in both scenarios, the targeted testing was still more cost effective than routine testing in Washington, DC. Overall, although the cost-effectiveness ratios were found to be higher for routine testing rather than targeted testing, all of our results were well below estimated lifetime costs of an HIV patient, (e.g. $380,000 in 2010 dollars [20]. Similarly the ICER for routine testing ($179,784) was relatively low.

## Discussion

This analysis of the cost effectiveness of routine versus targeted testing in a jurisdiction with a high HIV prevalence and a combined approach to HIV testing implementation found that targeted testing yields a higher proportion of HIV-positive persons previously unaware of their infections. However, we also found that routine testing averted nearly twice as many transmissions than targeted testing, thus lowering the chances of an infected but undiagnosed person unknowingly transmitting virus to others. Data from DC HIV surveillance suggest that these combined strategies have in fact been successful in reducing new infections and transmission as the number of new diagnoses has decreased by 42% and incident infections have declined 29% [10].

We found that routine testing and labor costs were substantially higher than targeted testing costs. A closer look at these costing components shows that clinic-based routine testing was the main driver of these costs. These higher costs may be explained by the fact that clinics tend to have a more highly specialized mix of health workers to administer tests and thus their staffing costs may exceed those of CBOs. Compared to clinics, hospital costs may be lower given the larger infrastructure available in which to integrate testing. In the future, program costs may experience further reductions due to expanded third party coverage in clinics and hospitals for HIV testing with the US Preventive Services Task Force making routine HIV screening a Grade A recommendation and with the implementation of the Affordable Care Act [21, 22]. These changes could justify the continued reliance on routine testing if payers are able to increase coverage in clinical settings where averted transmissions are highest.

Cost effectiveness analyses found that the costs per diagnosis and transmission averted were more than three times lower using targeted testing compared to routine testing. Moreover, CBO-based testing demonstrated superior cost effectiveness in both testing scenarios despite lower numbers of transmissions averted for routine testing and higher costs for targeted testing. The differences observed by testing site suggest that the cost effectiveness differences when looking at both approaches overall are due mostly to higher costs associated with routine testing at clinics, where the bulk of all testing occurred. For both approaches, not only were CE ratios (per new diagnosis and per averted submission) lower at CBOs but for CBOs, routine testing was actually more cost-effective than targeted testing (a 24.39% to 24.41% reduction in CE ratios).  

Our analyses are consistent with prior studies which sought to estimate costs associated with implementation of the 2006 CDC testing recommendations. Focusing on detailed program accounts we find a wide range of estimates in a variety of settings, with targeted testing appearing to be considerably more cost-effective than routine testing. Costs per HIV-infected person diagnosed in sexually transmitted disease clinics, emergency departments urgent care settings, and among CBOs using routine testing approaches have ranged from ~$1600 to $16,985 [19, 23–26]. Therefore our cost of $7,753 for routine testing falls near the mid-point of these estimates.

Our findings are also congruent with earlier national studies such as Holtgrave’s 2007 analysis that concluded that targeted testing was a lower cost option than routine opt-out testing [16]. Although from a strict cost-effectiveness perspective, targeted testing would be recommended, a number of issues should be noted. First, lifetime HIV treatment costs for an infected individual have been estimated to be as high as $380,000 in 2010 dollars, well above the cost per averted transmission for routine testing [20]. Thus, although targeted testing is the preferred strategy from a narrow cost-effectiveness perspective, routine testing still passes the cost-effectiveness criteria while having the bigger reach; thus the policy tradeoffs must be considered. For example, while targeted testing identified 239 new diagnoses, routine testing identified 461 new diagnoses. One could therefore argue that without routine testing, 222 infected persons may have been missed, despite it not being the most cost-effective approach in our analyses. Second, within each program type there is substantial variation in cost effectiveness across site types conducting testing. Most notably, CBOs are the most effective in delivering both forms of testing, compared with more clinical settings, i.e. clinics or hospitals. We attribute this to higher labor incurred in clinical settings (Table B in S1 File). It is also of note that when CBOs are considered separately, routine testing becomes the most cost-effective alternative overall. Nevertheless, inclusion of CBOs in implementation of rapid testing is essential in being able to conduct HIV testing among some of the highest risk populations. CBOs are often able to access marginalized or hard to reach populations including those who may be uninsured or who do not seek medical care regularly and might otherwise be missed in clinics or in hospital settings [27]. In fact, the higher prevalence of HIV among populations served by CBOs may account for their better cost effectiveness. Finally, while the payer’s perspective includes direct costs of health-care delivery, the societal perspective includes not only direct costs of health-care delivery but also costs to patients, employers and society as a whole. Therefore, all estimates presented here tend to be relatively conservative with respect to the general efficacy of testing.

Limitations of this analysis include the limited generalizability of our findings, assumptions regarding reductions in risky behavior, and absence of costs related to linkage to care. For example, the sheer nature of targeted testing compared to routine testing results in the identification of individuals who may differ in their behaviors and their access to testing locations, thus potentially resulting in the identification of more infected persons from targeted testing. To address potential bias in our assumptions regarding risk behaviors, we conducted a sensitivity analysis to assess the impact of different levels of transmission rates and found that targeted testing remained more cost effective than routine testing in Washington, DC. Despite these limitations, we believe that our findings may be applicable to other high prevalence areas, that reductions in risky behavior after diagnosis are well documented [28], and that future research can include the costs of linkage to care programs in Washington, DC such as the DC DOH Red Carpet Entry Program which links persons to care within 72 hours of identification [29]. Ongoing research to incorporate the costs of newer testing technologies and changes in test prices will further enhance our understanding of the cost effectiveness of these two testing strategies.

In conclusion, our study suggests that organizations in Washington, DC have been effective in scaling up both routine and targeted testing and have successfully identified large numbers of HIV-infected persons. As per CDC guidance, given the high prevalence rates in DC, a combination approach to HIV testing should continue to be supported. This combined approach allows for maximal identification of HIV-infected persons across a variety of settings. For clinics, while the costs of routine testing are high, this may be outweighed by the number of averted transmissions and the changing landscape for coverage of routine HIV testing. In the longer term, incorporating routine HIV testing into standard blood draws that does not rely on rapid testing may be more sustainable and cost-effective whereas the availability of rapid testing technologies allows CBOs to be able to conduct testing where there may be less infrastructure in place. Furthermore, for CBOs, a combination of routine and targeted testing is optimal with respect to costs per diagnosis and averted transmission and capitalizes on CBOs’ abilities to access communities that clinics and hospitals may not. Establishing partnerships between CBOs and clinics and hospitals may also be worth consideration as clinical settings can facilitate linkage to care among newly persons diagnosed through CBOs. Finally, as we get closer to meeting the goals of the National HIV AIDS Strategy and the number of new infections declines over time, follow-up analyses to assess the impact on testing approaches and costs will be warranted.

## Supporting Information

S1 File Tables A-E. **Table A.** This table provides additional detail regarding the testing costs based on the type of testing performed at a site. **Table B.** This table provides additional detail on the average hours and wages of pre-test counseling, HIV antibody testing, and post-test counseling, by type of testing site (clinic, hospital or community based organization). **Table C.** This table illustrates the findings from the cost-effectiveness analysis when Western Blot test results are incorporated into the model. Targted testing continues to have a lower cost per transmission averted with an incremental cost ratio of $178,328. **Table D.** This table illustrates the findings from the cost-effectiveness analysis when Western Blot test results are incorporated into the model and sites are further stratified by type of testing site (clinic, hospital or community based organization). With the addition of Western Blot testing, clinic based testing had the highest cost per averted transmission, mostly likely due to the high labor costs. **Table E.** This table highlights the findings from a senstivity analysis which compared differing HIV tranmission rates by testing approach. The main cost effectiveness analysis of routine and targeted testings used a simple weighted average transmission rate for test takers of both routine and targeted testing, which is 2.72% (= 0 X 84% + 17 X 16%). However, newly identified persons living with HIV who underwent targeted testing may engage in risky behaviors more than those who tested through routine testing. Hence, in this table, we doubled the portion of people who engage in risky behaviors, and found a higher transmission rate of 5.44% (= 0 X 68% + 17 X 32%). When the portion of people with risky behaviors is doubled, the cost per averted transmission is $53,091, which is 57.1% higher than the previous cost effectiveness ratio for targeted testing. In this scenario, the targeted testing is still more cost effective than routine testing in Washington, DC.
(DOCX)
