# Supplementary material for: Comparing Cost-Effectiveness of HIV Testing Strategies: Targeted and Routine Testing in Washington, DC
Source: PLoS One. 2015 Oct 14;10(10):e0139605. doi: 10.1371/journal.pone.0139605 (PMC4605630; doi:10.1371/journal.pone.0139605)
Supplement: S1 File — Table A. This table provides additional detail regarding the testing costs based on the type of testing performed at a site. Table B. This table provides additional detail on the average hours and wages of pre-test counseling, HIV antibody testing, and post-test counseling, by type of testing site (clinic, hospital or community based organization). Table C. This table illustrates the findings from the cost-effectiveness analysis when Western Blot test results are incorporated into the model. Targted testing continues to have a lower cost per transmission averted with an incremental cost ratio of $178,328. Table D. This table illustrates the findings from the cost-effectiveness analysis when Western Blot test results are incorporated into the model and sites are further stratified by type of testing site (clinic, hospital or community based organization). With the addition of Western Blot testing, clinic based testing had the highest cost per averted transmission, mostly likely due to the high labor costs. Table E. This table highlights the findings from a senstivity analysis which compared differing HIV tranmission rates by testing approach. The main cost effectiveness analysis of routine and targeted testings used a simple weighted average transmission rate for test takers of both routine and targeted testing, which is 2.72% (= 0 X 84% + 17 X 16%). However, newly identified persons living with HIV who underwent targeted testing may engage in risky behaviors more than those who tested through routine testing. Hence, in this table, we doubled the portion of people who engage in risky behaviors, and found a higher transmission rate of 5.44% (= 0 X 68% + 17 X 32%). When the portion of people with risky behaviors is doubled, the cost per averted transmission is $53,091, which is 57.1% higher than the previous cost effectiveness ratio for targeted testing. In this scenario, the targeted testing is still more cost effective than routine testing in Washington, DC. (DOCX) [file pone.0139605.s001.docx]

# Supporting Information File 1

# Table A. Testing Costs

|  | **Oral** | **Blood** | **Fingerstick** | **Oral and finger** |
| --- | --- | --- | --- | --- |
| unit cost of test ($) | 11 | 7.5 | 7.5 | 18.5 |
| unit cost of control  ($ per 100 tests) | 15.25 | 0 | 0 | 15.25 |

Source: Department of Health (DOH), 2011

# Table B. Labor Inputs: Average Hours and Wages

|  |  | **Clinics** | | **Hospitals** | | **CBOs** | |
| --- | --- | --- | --- | --- | --- | --- | --- |
|  |  | **(Counseling & Testing)** | | **(Testing)** | | **(Counseling & Testing)** | |
|  |  | **HIV +** | **HIV -** | **HIV +** | **HIV -** | **HIV +** | **HIV -** |
|  |  |  |  |  |  |  |  |
| Pretest Counseling | |  |  |  |  |  |  |
| Hourly Wage, pretest counseling^1^ | | 24.77 | 24.77 | 24.77 | 24.77 | 18 | 18 |
| Hours , pretest counseling^2,3^ | | 0.4 | 0.4 | 0 | 0 | 0.4 | 0.4 |
| Hourly Wage, collecting specimen^1^ | | 24.77 | 24.77 | 24.77 | 24.77 | 18 | 18 |
| Hours, collecting specimen^2,3^ | | 0.14 | 0.05 | 0.14 | 0.05 | 0.14 | 0.05 |
| Hourly Wage, administrative work^4^ | | 21.07 | 21.07 | 21.07 | 21.07 | 18 | 18 |
| Hours, administrative work^2,3^ | | 0.25 | 0.25 | 0 | 0 | 0.25 | 0.25 |
|  |  |  |  |  |  |  |  |
| HIV antibody testing | |  |  |  |  |  |  |
| Hourly Wage, lab technician, rapid test^5^ | | 21.52 | 21.52 | 21.52 | 21.52 | 18 | 18 |
| Hours, lab technician, rapid test^2,3^ | | 0.06 | 0.06 | 0.06 | 0.06 | 0.06 | 0.06 |
| Hourly Wage, lab technician, WB test^6^ | | 21.52 | 21.52 | 21.52 | 21.52 | 21.52 | 21.52 |
| Hours, lab technician, WB test^2,3^ | | 0.17 | 0.00 | 0.17 | 0.00 | 0.17 | 0.00 |
|  |  |  |  |  |  |  |  |
| Posttest counseling | |  |  |  |  |  |  |
| Hourly Wage, posttest counseling^1^ | | 24.77 | 24.77 | 24.77 | 24.77 | 18 | 18 |
| Hours, posttest counseling^2,3^ | | 0.55 | 0.185 | 0.24 | 0.02 | 0.55 | 0.185 |
| Hourly Wage, administrative work^4^ | | 21.07 | 21.07 | 21.07 | 21.07 | 18 | 18 |
| Hours, administrative work^2,3^ | | 0.25 | 0.25 | 0.25 | 0.25 | 0.25 | 0.25 |
|  |  |  |  |  |  |  |  |
| Total | | 42.44 | 27.56 | 19.59 | 8.29 | 33.32 | 21.51 |

Notes: 1) Department of Labor, Bureau of Labor Statistics. Occupational employment and wages, Washington-Arlington-Alexandria area (DC-VA-MD-WV). May 2012: 21-1011, substance abuse and behavioral disorder counselors: mean hourly wage. [http://data.bls.gov/oes/search.jsp?data_tool=OES].

2) Silva A, Glick NR, Lyss SB, Hutchinson AB, Gift TL, Pealer LN, Broussard D, Whitman S. Implementing an HIV and sexually transmitted disease screening program in an emergency department. Ann Emerg Med 2007 5;49(5):564-72.

3) Farnham PG, Hutchinson AB, Sansom SL, Branson BM. Comparing the costs of HIV screening strategies and technologies in health-care settings. Public Health Reports 2008, 123(Suppl 3), 51-62.

4) Department of Labor, Bureau of Labor Statistics. Occupational employment and wages, Washington-Arlington-Alexandria area (DC-VA-MD-WV). May 2012: 43-9199, office and administrative support workers, all other: mean hourly wage. [http://data.bls.gov/oes/search.jsp?data_tool=OES].

5) Department of Labor, Bureau of Labor Statistics. Occupational employment and wages, Washington-Arlington-Alexandria area (DC-VA-MD-WV). May 2012: 29-2012, Medical and Clinical Laboratory Technicians: mean hourly wage. [http://data.bls.gov/oes/search.jsp?data_tool=OES].

6) Confirmatory western blot tests for CBOs were performed in hospitals, therefore lab technicians’ hourly wages were higher for western blot test than rapid testing.

**Table C. Cost Effectiveness of Routine and Targeted HIV Testing including Western Blot tests, Washington, DC, 2011**

| **Measure** | **Routine** | **Targeted** |
| --- | --- | --- |
|  | **HIV testing** | **HIV testing** |
| a. number of tests^1^ | 104,424 | 17,932 |
| b. number testing positive, unique | 497 | 328 |
| c. number testing positive, aware | 36 | 89 |
| d. number testing positive, unaware | 461 | 239 |
| e. portion of number testing positive, unaware | 0.44% | 1.33% |
| f. transmission rate from unaware HIV + | 10.20% | 10.20% |
| g. transmission rate from aware HIV + | 2.72% | 2.72% |
| h. number of transmissions averted^2,3^ | 34.48 | 17.88 |
| i. testing costs ($) | 1,401,192 | 212,477 |
| j. labor cost ($) | 2,193,034 | 391,510 |
| k. total cost ($) | 3,594,226 | 603,987 |
| l. cost per new diagnosis ($) | 7,797 | 2,527 |
| m. cost per averted transmission ($) | 104,232 | 33,785 |
| Incremental CE ratio (ICER), per averted transmission | $178,328 | |
| **Note**: 1) Costs for the confirmatory Western Blot tests are included; Mean price per test in sites represented in our data was $38.50. 2) The number of averted HIV transmissions was estimated by multiplying the number of persons with HIV who became aware of their status and the difference in transmission rates before and after knowing their HIV status. h = d * [f - g]. 3) The average HIV transmission rate for all groups was used for the number of averted transmissions.  **Source**: Program Evaluation and Monitoring System (PEMS), Fiscal year 2011 Washington, DC DOH | | |

**Table D. Cost Effectiveness of Routine and Targeted HIV Testing including Western Blot tests by Site Type, Washington, DC, 2011**

| **Measure** | **Routine** | | | **Targeted** | | |
| --- | --- | --- | --- | --- | --- | --- |
|  | **CBO** | **Clinic** | **Hospital** | **CBO** | **Clinic** | **Hospital** |
| a. number of tests | 1,422 | 67,577 | 35,425 | 17,354 | 578 | 0 |
| b. number testing positive, unique | 25 | 311 | 161 | 324 | 4 | 0 |
| c. number testing positive, aware | 0 | 33 | 3 | 89 | 0 | 0 |
| d. number testing positive, unaware | 25 | 278 | 158 | 235 | 4 | 0 |
| e. portion of number testing positive, unaware | 1.76% | 0.41% | 0.45% | 1.35% | 0.69% | N/A |
| f. transmission rate from unaware HIV + | 10.20% | 10.20% | 10.20% | 10.20% | 10.20% | 10.20% |
| g. transmission rate from aware HIV + | 2.72% | 2.72% | 2.72% | 2.72% | 2.72% | 2.72% |
| h. number of transmissions averted | 1.87 | 20.79 | 11.82 | 17.58 | 0.30 | 0 |
| i. testing costs ($) | 16,388 | 957,972 | 426,832 | 209,942 | 2,535 | 0 |
| j. labor cost ($) | 30,746 | 1,866,693 | 295,595 | 383,433 | 8,077 | 0 |
| k. total cost ($) | 47,134 | 2,824,665 | 722,427 | 593,375 | 10,612 | 0 |
| l. cost per new diagnosis ($) | 1,885 | 10,161 | 4,572 | 2,525 | 2,653 | N/A |
| m. cost per averted transmission ($) | 25,205 | 135,838 | 61,127 | 33,757 | 35,468 | N/A |

Source: Program Evaluation and Monitoring System (PEMS), Fiscal year 2011 Washington, DC DOH

**Table E. Cost Effectiveness of Routine and Targeted HIV Testing Allowing for Different HIV Transmission Rates by Type of Testing, Washington, DC, 2011**

| **Measure** | **Routine** | **Targeted** | **Targeted** |
| --- | --- | --- | --- |
|  | **HIV testing** | **HIV testing** | **HIV testing** |
| **No Risky Behavior : Risky Behavior** | **1:1** | **1:1** | **1:2** |
| a. number of tests | 104,424 | 17,932 | 17,932 |
| b. number testing positive, unique | 497 | 328 | 328 |
| c. number testing positive, aware | 36 | 89 | 89 |
| d. number testing positive, unaware | 461 | 239 | 239 |
| e. portion of number testing positive, unaware | 0.44% | 1.33% | 1.33% |
| f. transmission rate from unaware HIV + | 10.20% | 10.20% | 10.20% |
| g. transmission rate from aware HIV + | 2.72% | 2.72% | 5.44% |
| h. number of transmissions averted^1,2^ | 34.48 | 17.88 | 11.38 |
| i. testing costs ($) | 1,401,191 | 212,477 | 212,477 |
| j. labor cost ($) | 2,193,034 | 391,510 | 391,510 |
| k. total cost ($) | 3,594,226 | 603,987 | 603,987 |
| l. cost per new diagnosis ($) | 7,797 | 2,527 | 2,527 |
| m. cost per averted transmission ($) | 104,232 | 33,785 | 53,091 |
| **Note**: 1) The number of averted HIV transmissions was estimated by multiplying the number of persons with HIV who became aware of their status and the difference in transmission rates before and after knowing their HIV status. h = d * [f - g]. 2) The average HIV transmission rate for all groups was used for the number of averted transmissions. 3) Costs for confirmatory Western Blot tests are included. | | | |
| **Source**: Program Evaluation and Monitoring System (PEMS), Fiscal year 2011 Washington, DC DOH | | | |
